# Brown Algae *Padina sanctae-crucis* Børgesen: A Potential Nutraceutical

**DOI:** 10.3390/md15100251

**Published:** 2017-09-26

**Authors:** Raquel B. S. S. Nogueira, Anna Cláudia A. Tomaz, Déborah R. Pessoa, Aline L. Xavier, João Carlos L. R. Pita, Marianna V. Sobral, Marcela L. C. Pontes, Hilzeth L. F. Pessôa, Margareth F. F. M. Diniz, George Emmanuel C. Miranda, Maria Aparecida R. Vieira, Marcia O. M. Marques, Maria de Fátima V. Souza, Emídio V. L. Cunha

**Affiliations:** 1 Postgraduate Program in Bioactive Natural and Synthetic Products, Health Sciences Center, Federal University of Paraíba, João Pessoa 58051-970, PB, Brazil; raquelbssn@gmail.com (R.B.S.S.N.); annacatomaz@gmail.com (A.C.A.T.); deborahribeiro11@gmail.com (D.R.P.); aline.lx@gmail.com (A.L.X.); joaocpita@gmail.com (J.C.L.R.P.); mariannavbs@gmail.com (M.V.S.); marcelalinspontes@gmail.com (M.L.C.P.); margarethdiniz.ufpb@gmail.com (M.F.F.M.D.); mfvanderlei@ltf.ufpb.br (M.d.F.V.S.); 2 Department of Pharmaceutical Sciences, Federal University of Paraíba, João Pessoa 58051-900, PB, Brazil; 3 Postgraduate Program in Development and Technological Innovation in Medicines, Health Sciences Center, Federal University of Paraíba, João Pessoa 58051-970, PB, Brazil; hilzeth@gmail.com; 4 Department of Molecular Biology, Federal University of Paraíba, João Pessoa 58051-900, PB, Brazil; 5 Department of Systematics and Ecology, Federal University of Paraíba, João Pessoa 58051-900, PB, Brazil; mirandag@dse.ufpb.br; 6 Center of R&D of Plant Genetic Resources, Agronomical Institute, CP28, Campinas 13001-970, SP, Brazil; mavieduc@ibb.unesp.br (M.A.R.V.); mortiz@iac.sp.gov.br (M.O.M.M.)

**Keywords:** Dictyotaceae, dolastane diterpene, phaeophytin, mannitol, palmitic acid, linolenic acid, cytotoxicity, genotoxicity, acute toxicity

## Abstract

*Padina sanctae-crucis* Børgesen is distributed worldwide in tropical and subtropical seas; belongs to the Dictyotaceae family, and has proven to be an exceptional source of biologically active compounds. Four compounds were isolated and identified, namely: dolastane diterpene new for the genus *Padina*; phaeophytin and hidroxy-phaeophytin new for the family Dictyotaceae, and; mannitol first described in this species. Saturated fatty acids as compared to the percentages of unsaturated fatty acids were shown to be present in greater abundance. Palmitic and linolenic acid were the main saturated and unsaturated acids, respectively. Cytotoxic and antioxidant activities were evaluated using human erythrocytes. In vivo evaluations of acute toxicity and genotoxicity were performed in mice. Methanolic extract of *P.* *sanctae-crucis* presented antioxidant activity and did not induce cytotoxicity, genotoxicity or acute toxicity. Since *Padina sanctae-crucis* is already used as food, has essential fatty acids for the nutrition of mammals, does not present toxicity and has antioxidant activity, it can be considered as a potential nutraceutical.

## 1. Introduction

The use of seaweed as food has strong roots in Asian countries such as China, Japan and the Republic of Korea, but demand for seaweed as food has now also spread to North America, South America, and Europe, as fresh or dried seaweed, or as an ingredient in prepared foods. It is also an ingredient for the global food and cosmetics industries, and is used as fertilizer and as an animal feed additive. The nutrient composition of seaweed varies, and is affected by species, geographic area, season, and water temperature [1]. 

Macroalgae are considered a food supplement for the 21st century, because they contain proteins, lipids, polysaccharides, minerals, vitamins, and enzymes. These sea vegetables are of nutritional interest because they are low calorie food, but rich in vitamins, minerals and dietary fiber [1].

Marine algae have been identified as rich sources of structurally diverse bioactive compounds with great pharmaceutical potential [2,3]. Many seaweed species have been used as herbal medicines in China to treat goiter, scrofula, urinary disease, dropsy, stomach ailments, and hemorrhoids [4].

Marine macroalgae have been the focus of structural investigations by natural product chemists due to previous studies showing that seaweeds present a great number of biological benefits to human health, including antimicrobial, cytotoxic, antimitotic, anticancer and antimutagenic activities [5,6].

Several alga species are known to produce a variety of toxic metabolites that can pose a threat to aquatic organisms, animals and humans. Moreover, these metabolites have been thought to cause serious diseases including certain cancers and neurodegenerative disorders [7].

In a human context, the potential negative impacts of oxidants are widely recognized, and both modern science and folk remedy utilization have responded by providing functional products that involve food, medicines and cosmetics. Consumption of products high in antioxidant compounds is thought to alleviate cellular stresses brought about by the influence of reactive oxygen species [8,9].

While antioxidant benefits associated with consuming various terrestrial plants have long been accepted, relatively little emphasis has been placed on the merits of consuming marine macroalgae for the same benefits [10]. Several compounds with anti-oxidative action have been isolated from brown algae, most of them belonging to their phenolic fractions [11]. Research is advancing into using marine algae to take advantage of their naturally occurring antioxidant compounds and other nutritive components [12,13].

*Padina sanctae-crucis* Børgesen belongs to kingdom Chromista, phylum Ochropyita, subphylum Phaeista, class Phaeophyceae, order Dictyotales and family Dictyotaceae [14]. The family Dictyotaceae belongs to the most well studied among the brown algae and has proven to be an exceptional source of biologically active compounds [15]. The genus *Padina* comprises approximately 52 species [16] distributed worldwide in tropical and subtropical seas. In the Pacific Ocean region, *Dictyota* sp. or the brown Padina algae, or sea fan ribbon weeds, are used as food dressings, or in soups and stews [17].

Considering the absence of data in the literature concerning Dictyotaceae family species, the aim of this work is to contribute to chemotaxonomic, pharmacological and toxicological study of *Padina sanctae-crucis* Børgesen, (*P. sanctae-crucis).*

## 2. Results

Compound **1** presented as yellowish-white crystals; with a melting point in the range of 220–222 °C. The IR spectrum indicated the presence of hydroxyl (3327 cm^−1^) groups [18]. Absorptions between 2960 and 2920 cm^−1^ were related to methyl, methylene and methine groups; and absorptions between 1456 and 1425 cm^−1^ were related to carbon double bonds, which together with the absorption at 896 cm^−1^ suggested 1,1-disubstituted alkene group. The ^13^C Attached-Proton-Test-NMR APT-NMR spectrum (CD_3_OD) showed 20 signals for diterpene nuclei, being six non-hydrogenated, three methine, seven methylene and four methyl carbons. The signals at δ_C_ 154.3 and 109.5 were assigned to C-1 and C-15, characterizing exocyclic double bonds from dolastane-type diterpenes. Signals at δ_C_ 42.3, 47.0, 27.8, 20.0 and 24.5 reinforce these data. According to literature data [19], the signals at δ_C_ 20.0 (CH_3_-16) and 24.5 (CH_3_-20) refer to dolastane diterpene methyls, and signals at δ_C_ 81.1, 82.1 and 87,0 to carbons bonded to hydroxyl groups, being, respectively, C-4, C-9 and C-14. The ^1^H-NMR spectrum (CD_3_OD) showed a broad singlet (δ_H_ 3.43, (brs, H-4)) and a double doublet (δ_H_ 5.60 (dd, *J* = 4 5 and 13.5 Hz), H-7), characterizing protons bonded to hydroxylated carbons and olefinic protons, respectively. According to literature, the two singlets at δ_H_ 0.81 (H-16), 1.22 (H-20) refer to methyl protons, respectively, CH_3_-16 and CH_3_-20. The CH_3_-20 signal at δ_H_ 1.22 is consistent with the insertion of a hydroxyl at C-9 (δ_C_ 87.0) [20] with β-orientation. Direct correlations between protons and carbons were observed through the 2D NMR data of the Heteronuclear multiple-quantum correlation spectroscopy (HMQC) experiment and are compiled in Table 1. The 2D NMR data of the Heteronuclear Multiple Bond Correlation (HMBC) experiment confirmed the insertion of a hydroxyl group at C-4 through the ^2^*J* correlation between the proton at δ_H_ 0.81 (CH_3_-16) and C-5 (δ_C_ 43.3) and its ^3^*J* correlations with C-4, C-6 and C-14 (respectively, δ_C_ 81.1, 32.5 and 82.1). In addition, the presence of a double bond between C-7 and C-8 was strengthened by the ^3^*J* correlations between the proton at δ_H_ 1.22 (CH_3_-20) and C-8, C-11, and C-13 (respectively, δ_C_ 156.2, 42.3 and 45.0) and its ^2^*J* correlation with C-12 (δ_C_ 47.0). Other correlations are shown in Table 1. The 2D NMR data from the COSY experiment established correlations between vicinal and geminal protons as can be seen in Table 1. The 2D NMR data of the NOESY experiment confirmed the cis orientation of the protons that correlate at δ_H_ 0.81 (CH_3_-16) and δ_H_ 3.43 (H-4). Further, spatial correlations between the protons at δ_H_ 1.22 (CH_3_-20) and δ_H_ 1.67 (H-6), as well as at δ_H_ 1.99 (CH-17) and δ_H_ 1.67 (CH_2_-6) with δ_H_ 5.60 (H-7) were observed. Spectral data compilation and comparison to the data in the literature [9] showed that compound **1** was identifiable as the dolastane diterpene (4*S*, 9*R*, 14*S*)-4,9,14-trihydroxydolasta-1(15),7-diene, the first reported in the genus *Padina*.

**Figure 1:**
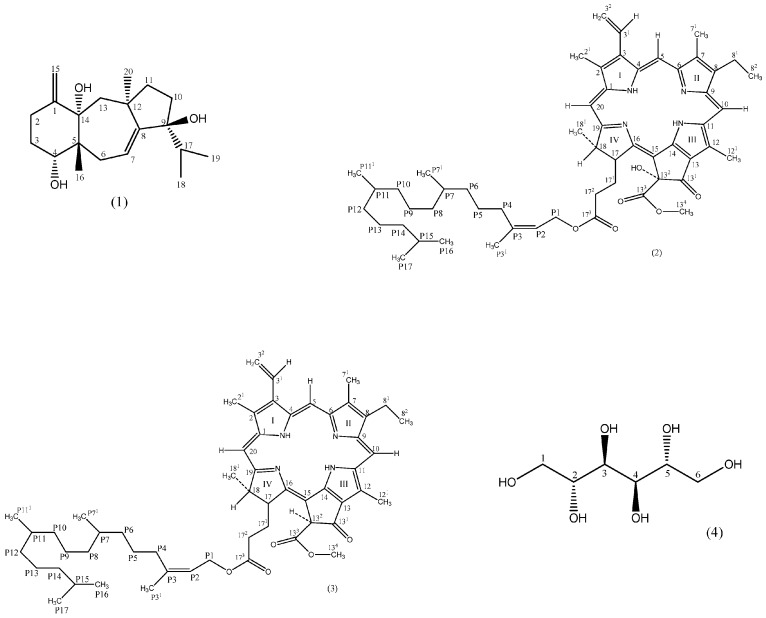
Structures of compounds **1**–**4**.

Using similar methods to those described above, compounds **2**–**4** were identified as phaeophytin (2) [21,22], and 13^2^-hydroxy-(13^2^-*S*)-phaeophytin a (3) [23], and mannitol (4) [24,25,26], with compounds **2** and **3** new for the family Dictyotaceae and compound **4** first described in this species (Figure 1).

The analysis of the 1/8 trans-esterified fraction from the hexane phase of the *P. sanctae-crucis* ethanol extract [27] under GC-MS allowed identification of 89.82% of unsaponifiable lipids, which were identified as: myristic acid (6.02%); palmitic acid (68.84%); linoleic acid (0.73%); oleic acid (1.61%); linolenic acid (9.75%), and; stearic acid (2.87%). Saturated fatty acids were shown to be present in greater abundance (77.73%) as compared to the unsaturated fatty acids percentage (12.09%) (Table 2). 

**Figure 2:**
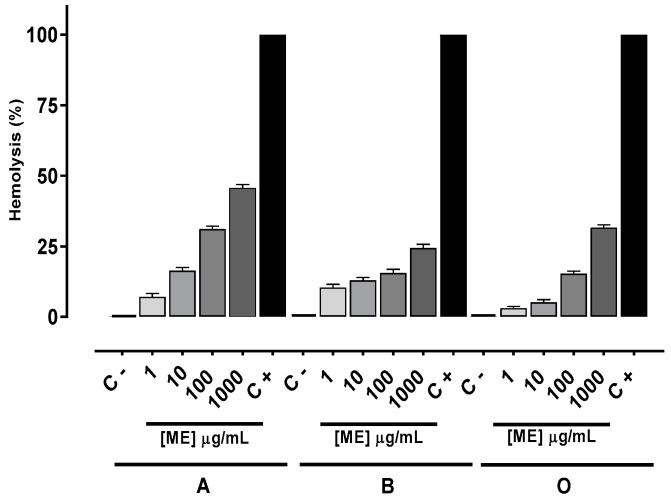
Hemolytic activity of methanolic extract (ME) on human erythrocytes of blood groups A, B and O. Values are means ± standard error of mean (s.e.m.) from three independent experiments. C − (erythrocytes), and C + (erythrocytes + Triton X-100).

To evaluate the in vitro toxicity of the methanolic extract (ME), hemolytic assays with human erythrocyte blood groups ABO were performed. The hemolysis percentage increased in a concentration-dependent manner. However, after treatment with ME at a concentration of 1000 μg/mL none of the three blood groups reached 100%. The hemolytic effect to human blood groups A, B and O, at a concentration of 1000 μg/mL, was respectively 45.4%, 24.3% and 30.6% (Figure 2).

**Figure 3:**
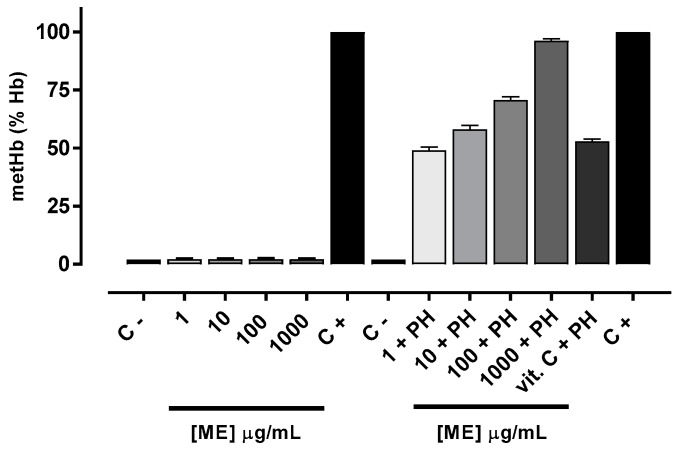
Oxidant and antioxidant effect of ME in human erythrocytes. Values are means ± standard error of mean (s.e.m.) from three independent experiments. C − (erythrocytes) and C + (erythrocytes + PH).

The oxidant potential was evaluated considering the amount of methemoglobin formed after exposure to ME. The antioxidant potential was measured by effectiveness of ME in inhibiting methemoglobin formation after phenylhydrazine (PH) exposure. As depicted in Figure 3, the amount of methemoglobin formed in the absence and presence of various concentrations of ME were the same, indicating no oxidizing effect. In contrast, the methemoglobin formation was significantly reduced in erythrocytes pretreated with ME in a concentration-dependent manner. It is interesting to note that the erythrocyte antioxidant protection conferred by ME at a concentration of 1 μg/mL (47.6%) and 10 μg/mL (57.9%) was similar the protective effects afforded by Vitamin C (52.7%) [28] (Figure 3).

To investigate possible in vivo toxic effects of the ME an acute toxicological study was conducted. With administration of the ME, the animals showed no behavioral changes. All male and female mice remained alive after 14 days of observation. 

Table 3 contains values relating to water, feed consumption and weight gain as evaluated during the 14 days of observation. According to the results obtained, after administration of a single dose of ME (2000 mg/kg), no significant differences were observed in water and feed consumption for either male and female groups, as compared to the control group. The body weight of the animals treated with the extract also did not present significant alteration. 

Table 4 contains values of the organ indices evaluated after treatment with ME. No significant changes were observed between mice treated with ME or controls.

Toxicological analyses of the effects of ME also included assessment of biochemical and hematological parameters. 

Table 5 contains values for the biochemical parameters evaluated after treatment with ME. No significant changes were observed between ME treated mice and the control.

As for hematological evaluations, a significant increase in the female erythrocyte counts when treated with ME as compared to the control group was observed (Table 6). However, despite the observed change in the total number of erythrocytes, it was within the normal range observed for mice [29]. 

The parameters mean corpuscular volume (MCV), mean corpuscular hemoglobin (MCH) and mean corpuscular hemoglobin concentration (MCHC) did not change after the treatment with ME. As for the differential leukocyte count, leukopenia and lymphocytosis were observed in the females treated with ME when compared to the control group (Table 6). 

To evaluate the possible in vivo genotoxic effects of the ME, we performed micronucleus assaying; the results are presented in Table 7. Treating the animals with the extract did not induce an increase in the micronucleated erythrocytes frequency in the peripheral blood as compared to the control group (5% Tween 80), suggesting no evidence of genotoxicity.

## 3. Discussion

This study led to the isolation and identification of the compounds (4*S*, 9*R*, 14*S*)-4,9,14-trihydroxydolasta-1(15),7-diene (1), phaeophytin (2), 13^2^-hydroxy-(13^2^-*S*)-phaeophytin (3), and mannitol (4), with compound **1** new for the genus *Padina*, compounds **2** and **3** new for the family Dictyotaceae, and compound **4** first described in this species. Fatty acids were also identified with the percentage of saturated acids being in greater abundance than unsaturated acids. The major components of the saturated and unsaturated fatty acid percentages were respectively palmitic and linolenic acid. 

Palmitic acid increases stability of fixed oils against peroxidation, and contributes to the production of various types of margarines when mixed into the oil in the proportion of 15–25% [30]. Unsaturated fatty acids are of great interest in the cosmetics and biotechnology industries [31]. They have effects on health in various processes such as: regulation of plasma lipid levels; cardiovascular and immune functions; neuronal development; eye function; composition and function of membranes; eicosanoid synthesis; cellular signaling; regulation of gene expression [32]; and even depression [33]. In mammalian nutrition, linoleic and linolenic acids are essential fatty acids [34]. 

In cytotoxicity assays, the mechanical stability of the erythrocyte membrane is a good indicator of in vitro damage, since drugs can alter this delicate structure [35]. Damage to red cells also provides a preliminary model to study either protective or toxic effects of substances or conditions associated with oxidative stress [36,37,38,39]. 

The negligible hemolytic effect induced by the ME at all concentrations tested indicates that no damage occurred in the cell membrane of most of the cells and, thus, these have low cytotoxicity. The ABO blood groups were determined by adding different sugars to a protein on the surface of the erythrocytes [40], which contributes to explaining the different hemolytic effect of ME in each blood type.

The ME presented antioxidant effect similar to that of vitamin C, a proven antioxidant agent. Due to concerns about the toxic and carcinogenic effects of synthetic antioxidants [41,42], there is an increasing interest in antioxidants from natural sources [43]. Many epidemiological reports suggest protective effects against atherosclerosis, neuronal degeneration, cancer, rheumatoid arthritis, diabetes mellitus, inflammation and vascular disease [44,45,46]. Reactive oxygen species are toxic since they can oxidize biomolecules, leading to cell death and tissue injury [47]. 

Human erythrocytes can be used through in vitro experimental models to investigate the antioxidant potential of vegetable extracts. It must be kept in mind, however, that such antioxidant activities found in erythrocytes do not necessarily reflect the antioxidant defenses of the whole organism. In addition, there are genetic variations between individuals that result in altered gene expression, leading to varied and potentially undesired effects in the antioxidant defenses [28].

Antioxidant activity has been reported in several species of *Padina*; among them we can mention: *P. minor* [48]; *P. pavonica* [49]; *P. gymnospora* [50]; *P. boergesenii* [51]; *P*. *tetrastomatica* [52], and; *P*. *australis* [53].

Throughout the acute toxicological study, the ME did not induce significant differences or changes in behavior, water and feed consumption, body weight, the organ indexes (heart, liver, kidneys, thymus, spleen), biochemical parameters (aspartate aminotransferase, alanine aminotransferase, urea, creatinine), or death. As to the feminine hematological parameters, a significant increase in erythrocyte and lymphocytes counts, and a decrease in total leucocytes, was observed. However, all these changes are within the normal range for female mice [29]. In male mice, we noted leukocytosis, characteristic of a defense response of the immune system.

Treatment of the animals with the ME did not induce micronucleated erythrocyte frequency increases in peripheral blood, thus suggesting no evidence of genotoxicity. 

A nutraceutical is any substance that is a food or a part of food and provides medical or health benefits, this includes use for both prevention and treatment of disease. Such products may range from isolated nutrients, dietary supplements and specific diets, to genetically engineered designer foods and herbal products [54]. To develop nutraceuticals and pharmaceutical compounds from marine source, both price and sufficient supply are critical. Among the marine algae, brown algae is a promising candidate for the development of functional foods, since mass production is relatively easy through aquaculture [55]. Brown algae account for approximately 59% of the total macroalgae cultivated in the world and can be cultivated on seashores in large scales. Their growth rate is relatively rapid, and their bioactive compounds such as proteins, polyphenols and pigments can be controlled during production by manipulating the culture conditions [56].

Since *Padina sanctae-crucis* is used as food, has essential fatty acids for the nutrition of mammals, does not present toxicity, and has antioxidant activity, it can be considered as a potential nutraceutical.

## 4. Materials and Methods 

### 4.1. General

Silica gel 60 (Merck, Darmstadt, Germany 7734 (0.063-0.2 mm particle, 70-230 mesh) and Sephadex LH-20 (GE Healthcare, Stockholm, Sweden) were used for column chromatography. The melting points of the constituents were tested on a MQAPF-302 apparatus (Microquímica Equipamentos Ltda., São Paulo, Brazil). The IR spectra were recorded on a FT-IR-1750 Perkin-Elmer spectrometer (Waltham, MA, USA). ^1^H (500 MHz), and ^13^C (125 MHz) NMR spectra were recorded on Varian 500 NRM-System and Brucker-AC 500 spectrometers (Billerica, MA, USA). GC-MS analysis was carried out using a Shimadzu QP-5000 GC-MS system (Redmond, WA, USA), operating in electron ionization mode at 70 eV.

### 4.2. Collection, Extraction and Iolation 

The brown alga *Padina sanctae-crucis* was collected in the coastal region of Bessa (07°04′33′′S and 034°49′31′′W), João Pessoa, Paraiba, Brazil, in November 2009. The specimen was identified by Dr. George Emmanuel Cavalcanti de Miranda. Three voucher specimens (JPB 40017, JPB 40018 and JPB 40019) are deposited at the Lauro Pires Xavier Herbarium of the Federal University of Paraíba, Brazil.

Two experiments were then carried out: 

In the first, the dried material (1 kg) was extracted with 95% EtOH (4.0 L) for 72 h at room temperature. The EtOH extract was concentrated under reduced pressure to give 185.75 g of the crude ethanol extract (CEE), which was solubilized in EtOH:H_2_O (7:3), yielding the hydroalcoholic solution. This was then partitioned with hexane, dichloromethane, ethyl acetate and n-butanol, providing 6.21 g of hexane phase, 12.98 g of dichloromethane phase, 114.2 mg of EtOAc phase and 9.03 g n-BuOH phase. The solvents were used alone and in increasing order of polarity.

In the second, the alga was subjected to cold extraction with hexane, dichloromethane, ethyl acetate and methanol; yielding the following extracts: hexane (1.27 g), dichloromethane (3.04 g), ethyl acetate (2.21 g) and methanol (113.34 g). A precipitate was separated from the hexane extract resulting in the isolation of compound **1** in the form of yellowish-white crystals (17.0 mg). The ethyl acetate extract (2.21 g) was subjected to column chromatography with silica gel 60 and the eluents hexane, ethyl acetate and methanol alone or in mixtures, affording 61 fractions of 50 mL each which were concentrated under reduced pressure, analyzed through analytical thin layer chromatography (TLC), and combined according to their retention factors (Rf’s). The subfraction 27/51 (97.4 mg) was subjected to preparative TLC, using a mixture of hexane and ethyl acetate in the ratio of 7:3 as eluent; resulting in the isolation and purification of two bluish green amorphous solids, compounds **2** (15.0 mg) and **3** (25.6 mg).

Three (3 g) of the methanolic extract (ME) was subjected to column chromatography using Sephadex LH-20 and the methanol eluent gave 40 fractions of 50 mL each which were concentrated under reduced pressure, and analyzed through analytical TLC. Subfraction 5/22 was again subjected to chromatography following the same methodology, providing 23 subfractions of 50 mL which were concentrated, analyzed and combined, following the previously adopted methods. Subfraction 5/13 yielded a supernatant and 21.2 mg of a white solid precipitate, compound **4**.

Coumpound **1**: Yellowish-white crystals, M.P.: 220–222 °C, ^1^H-NMR and ^13^C-NMR: See Table 1.

Coumpound **2**: Dark green powder; IR (KBr) V_máx_ (cm^−1^): 3452 (N–H), 2963, 2854 (–OCH_3_), 1377 (C–N), 1739 (–O–C=O), 1697 (C=O), 1620 (–O–C=O); ^1^H-NMR (CDCl_3_, 500 MHz): δ 9.51 (s, H-10), 9.35 (s, H-5), 8.60 (s, H-20), 7.95 (dd, *J =* 17.85 and 11.48 Hz, H-3^1^), 6.30 (s, H-13^2^), 6.27 (*trans*) (d, *J* = 17.95 Hz, H-3^2^a), 6.18 (*cis*) (d, *J* = 11.10 Hz, H-3^2^b), 4.34 (m, H-18), 4.15 (m, H-17), 3.91 (s, H-13^4^OCH_3_), 3.69 (s, H-12^1^), 3.63 (m, H-8^1^), 3.39 (s, H-2^1^), 3.19 (s, H-7), 1.84 (d, H-18^1^), 1.66 (m, H-8^2^); ^13^C-NMR (CDCl_3_, 125 MHz): δ 189.81 (C-13^1^), 173.18 (C-17^3^), 172.63 (C-19), 169.77 (C-13^3^), 161.19 (C-16), 155.55 (C-6), 150.92 (C-9), 149.59 (C-14), 145.25 (C-8), 142.34 (C-1), 138.14 (C-11), 136.82 (C-3), 136.51 (C-4), 136.14 (C-7), 131.10 (C-2), 129.19 (C-3^1^), 129.14 (C-13), 129.03 (C-12), 123.11 (C-3^2^), 105.10 (C-15), 104.59 (C-10), 97.56 (C-5), 93.72 (C-20), 64.90 (C-13^2^), 53.07 (C-13^4^OCH_3_), 51.42 (C-17), 50.36 (C-18), 31.42 (C-17^2^), 29.89 (C-17^1^), 23.28 (C-18^1^), 19.60 (C-8^1^), 17.52 (C-8^2^), 12.32 (C-12^1^), 12.26 (C-2^1^), 11.35 (C-7^1^), 61.69 (C-P1), 117.93 (C-P2), 143.02 (C-P3), 39.98 (C-P4), 25.17 (C-P5), 37.57 (C-P6), 32.93 (C-P7), 37.50 (C-P8), 24.60 (C-P9), 36.82 (C-P10), 32.79 (C-P11), 37.44 (C-P12), 24.95 (C-P13), 39.53 (C-P14), 28.14 (C-P15), 22.89 (C-P16), 22.80 (C-P17), 19.84 (C-P111), 19.90 (C-P71), 16.48 (C-P31).

Compound **3**: Bluish-green powder; IR (KBr) V_máx_ (cm^−1^): 3452 (N–H), 2963, 2854 (–OCH3), 1377 (C–N), 1739 (–O–C=O), 1697 (C=O), 1620 (–O–C=O); ^1^H-NMR (CDCl_3_, 500 MHz): δ 9.58 (s, H-10), 9.46 (s, H-5), 8.60 (s, H-20), 8.01 (dd, *J* = 18.00 and 11.50 Hz, H-3^1^), 6,28 (*trans*) (d, *J* = 18.00 Hz, H-3^2^a), 6.17 (*cis*) (d, *J* = 11.50 Hz, H-3^2^b), 4.55 (m, H-18), 4.15 (m, H-17), 3.88 (s, H-13^4^OCH_3_), 3.71 (m, H-8^1^), 3.60 (s, H-12^1^), 3.46 (s, H-2^1^), 3.22 (s, H-7^1^), 1.68 (d, H-8^2^), 1.65 (m, H-18^1^); ^13^C-NMR (CDCl_3_, 125 MHz): δ 192.01 (C-13^1^), 172.78 (C-17^3^), 172.43 (C-19), 173.56 (C-13^3^), 162.51 (C-16), 155.35 (C-6), 151.06 (C-9), 149.88 (C-14), 145.21 (C-8), 142.04 (C-1), 137.85 (C-11), 136.32 (C-4), 136.25 (C-7), 136.07 (C-3), 131,74 (C-2), 129.10 (C-3^1^), 129.70 (C-13), 129.40 (C-12), 122.82 (C-3^2^), 107.71 (C-15), 104.24 (C-10), 97.92 (C-5), 93.62 (C-20), 89.00 (C-13^2^), 51.90 (C-13^4^OCH_3_), 51.76 (C-17), 50.35 (C-18), 31.65 (C-17^2^), 29.40 (C-17^1^), 23.65 (C-18^1^), 19.70 (C-8^1^), 17.40 (C-8^2^), 12.26 (C-12^1^), 12.07 (C-2^1^), 11.25 (C-7^1^), 61.53 (C-P1), 117.95 (C-P2), 143.69 (C-P3), 39.82 (C-P4), 25.02 (C-P5), 37.40 (C-P6), 32.76 (C-P7), 37.30 (C-P8), 24.42 (C-P9), 36.65 (C-P10), 32.62 (C-P11), 37.27 (C-P12), 24.76 (C-P13), 39.36 (C-P14), 27.95 (C-P15), 22.68 (C-P16), 22.64 (C-P17), 19.48 (C-P111), 19.70 (C-P71), 16.03 (C-P31).

Compound **4**: White powder; M.P.: 165–167 °C; IR (KBr) V_máx_ (cm^−1^): 3398.57–3290.56 (O–H), 2970–2910 (CH), 1082.07, 1020.34 (C–O); ^1^H-NMR (DMSO-d6, 500 MHz): δ 3.60 (td, *J* = 2.0; 6.0 and 10.0 Hz, 2H, H-1 and 6) and 3.38 (m, H-1 and 6), 3.45 (m, H-2,5), 3.54 (t, *J* = 7.5 Hz, 1H, H-3 and 4); 4.31 (t, *J* = 5.0 Hz, 1H, OH-1), 4.40 (d, *J* = 5.0 Hz, 1H, OH-2), 4.13 (d, *J* = 7.0 Hz, 1H, OH-3); ^13^C-NMR (DMSO-d_6_, 125 MHz): δ 69.04 (C-1 and 6), 74.93 (C-2 and 5), 76.54 (C-3 and 4).

### 4.3. Fatty Acids Transesterification

The methodology developed by Maia et al. (1993) [27] and Saastamoinen et al. (1989) [30] was adopted to perform trans-esterification of fatty acids from the 1/8 fraction of the hexane phase of the *P. sanctae-crucis* ethanol extract. The fatty acid methyl esters were prepared as follows: a test tube held the saponification process using 30 mg of the sample together with hydroxide solution sodium (0.5 N) in methanol (4 mL), followed by esterification with 5 mL of ammonium chloride, sulfuric acid and methanol at a ratio of 1:1 and 5:3, respectively. Shortly thereafter, 4 mL of saturated sodium chloride solution was added to the test tube under stirring for 30 s, and then 5 mL of hexane (brand Synth, lot 65741) was also added to the test tube for 30 s and stirring. The solution was then allowed to stand to complete separation of the respective phase. After separation, the upper phase (hexane phase) was transferred using a Pasteur pipette into a capped vial and kept under refrigeration until completion of the un-saponifiable lipids analyses, which were performed in a gas chromatograph coupled to a mass spectrometer (GC-MS, Shimadzu QP-5000) operating with an electron impact of 70 eV).

The GC-MS instrument was equipped with OV-5 capillary column, using a stationary phase composed by 5% phenyl and 95% dimethylpolysiloxane (Ohio Valley Specialty Chemical, Inc., 30.0 m × 0.25 mm × 0.25 μm), with helium carrier gas (1.7 mL/min), split: 1/30, injector at 220 °C and the detector at 230 °C. An aliquot of 250 μL of the hexane phase obtained through trans-esterification, consisting of fatty acid methyl esters, was diluted in 250 μL of hexane (HPLC grade), and 1 μL of this solution was injected along the following temperature program: 60–100 °C, 5 °C/min; 100–170 °C, 10 °C/min; 170 °C (2 min); 170–173 °C, 1.5 °C/min; 173–180 °C, 1 °C/min; 180 °C (7 min); 180–200 °C, 6 °C/min; 200 °C (20 min). The lipid substances were identified by comparative analysis of their mass spectra with the database system GC-MS (Nist 62.lib), the literature [31] and commercial standard samples.

### 4.4. Experimental Animals

Swiss mice of both sexes, 6–8 weeks of age with an average weight 28–32 g, were obtained from the Federal University of Paraíba (Paraíba, Brazil) and were used throughout the experiments. They were housed in single-sex cage conditions with a 12-h light/dark cycle, at constant temperature (21 ± 1 °C), with free access to water and pellet food. Six hours before each experiment, the animals received only water, to avoid test substance absorption interference with food. The experiments were performed after protocol approval by the Animal Studies Committee of Federal University of Paraíba (0509/109). 

### 4.5. Cytotoxicity Assay

Cytotoxic activity is directly related to the hemolytic effect induced by ME [57]. Briefly, human erythrocytes samples were obtained from blood to be discarded by the University Hospital Lauro Wanderley/UFPB Transfusion Unit. To obtain a suspension of erythrocytes, 1.5 mL of whole blood was then made up to 10 mL in NaCl 0.9%, and centrifuged at 3000 rpm for 5 min. The supernatant was then removed by gentle aspiration, and the above process was repeated two more times. Erythrocytes were finally re-suspended in NaCl 0.9% to make 0.5% suspension for the hemolysis assay. ME (at 1, 10, 100 and 1000 μg/mL), dissolved in DMSO (Vetec) (5%), was added to the suspension of erythrocytes and incubated at 100 rpm at 22 ± 2 °C under slow and constant agitation (100 rpm, for 60 min) and then centrifuged at 3000 rpm for 5 min. The absorbance of the supernatants was determined at 540 nm using a UV-Vis Spectrophotometer (UV-1650PC Shimadzu^®^) to measure the extent of erythrocyte lysis. A suspension of erythrocytes was used as a negative control (0% hemolysis), and the erythrocyte suspension plus 1% Triton X-100 (Vetec) (for 100% hemolysis). All the experiments were performed in triplicate and after approval of the protocol by the Ethics Committee in Research of Federal University of Paraíba (0306/11). 

### 4.6. Oxidant and Antioxidant Evaluation

The erythrocytes were washed twice as described above, and re-suspended in PBS (NaH_2_PO_4_·2H_2_O 123 mmol/L, Na_2_HPO_4_ 27 mmol/L, NaCl 123 mmol/L) supplemented with glucose (200 mg/dL) pH 7.6 to a final hematocrit of 35%. Human erythrocytes in suspension were treated with extracts (1, 10, 100 and 1000 μg) for 60 min at 22 ± 2 °C under slow (100 rpm) and constant agitation. The erythrocyte suspension was the negative control and the erythrocyte suspension plus phenylhydrazine 1 mmol/L (Sigma) was the positive control. The methemoglobin concentration was measured spectrophotometrically (630 nm) as the percentage of total hemoglobin (540 nm) to evaluate the oxidant potential. Afterwards the samples were aerated and exposed to phenylhydrazine (1 mmol/L) for a further 20 min under the same conditions. The methemoglobin concentration as a percentage of the total hemoglobin was measured to evaluate the antioxidant effect [20]. All the experiments were performed in triplicate.

### 4.7. Acute Toxicity Studies

For acute toxicity studies, 12 male and 12 female mice were divided into two groups (6 males or 6 females per group). The extract was administered by gavage to the mice at a dose of 2000 mg/kg, while the control group received vehicle alone. The general behavior of the mice and signs of toxicity were observed continuously for 1 h after the administration of the ME, then intermittently for 4 h, and thereafter over a period of 24 h [58]. The mice were further observed once a day, to 14 days, following the treatment for behavioral changes and signs of toxicity and/or death, and for latency to death. Body weights were measured at the beginning and end of the treatment. On day 14, peripheral blood samples from the controls and treated mice were collected from the retro-orbital plexus under light sodium thiopental anesthesia (40 mg/kg—i.p.). For biochemical analysis, the blood samples from the controls and treated mice were centrifuged, and the levels of urea, creatinine, alanine aminotransferase (ALT) and aspartate aminotransferase (AST) were determined. For the hematological analysis we used heparinized whole blood; for the hematological parameters: (hemoglobin level, erythrocyte count, hematocrit, red cell indices such as mean corpuscular volume (MCV), mean corpuscular hemoglobin (MCH), mean corpuscular hemoglobin concentration (MCHC), and total and differential leukocyte count) were performed. The animals were also sacrificed by cervical dislocation; the heart, kidneys, liver, spleen and thymus were excised and weighed for determination of organ indices (organ weight/body weight).

### 4.8. Micronucleus Test

For the micronucleus assay, a group of six Swiss mice males were orally treated with the dose of 2000 mg/kg. A positive control group (cyclophosphamide 50 mg/kg—i.p.) and a negative control group (saline and tween 80 at 5%) were included. After 24 h the animals were anesthetized with sodium thiopental (40 mg/kg—i.p.) and peripheral blood samples were collected by orbital plexus for preparation of the blood extensions. For each animal three blood extensions were prepared and a minimum of 2000 erythrocytes counted to determine the frequency of micronucleated erythrocytes [59].

### 4.9. Statistical Analysis 

The results obtained were analyzed with the software GraphPad Prism 5.0^®^ (GraphPad Prism Software, San Diego, CA, USA)and expressed in mean ± s.e.m. using unpaired *t*-test for two-column analysis, and one-way analysis of variance (ANOVA) for comparing more than two columns, followed by the Tukey’s test (parametric variables), or Dunnett (non-parametric) and the results were considered significant when *p* < 0.05.

## 5. Conclusions

The chemical study of *Padina sanctae-crucis* led to the isolation and identification of four secondary metabolites. Substances 1–3 are the first reported for the *Padina* genus and substance 4 is the first described in the species *P. sanctae-crucis*. The presence of saturated fatty acids whose major component was palmitic acid, and of unsaturated fatty acids, particularly linolenic acid, suggests that *P. sanctae-crucis* has great nutritional potential. The methanolic extract was able to prevent oxidative stress and did not present significant cytotoxicity, acute toxicity or genotoxicity in the experimental models evaluated. Since *Padina sanctae-crucis* possesses essential molecules for nutrition, is able to prevent oxidative stress and does not present significant toxicity, we consider it a potential nutraceutical.

## Figures and Tables

**Table 1:** ^1^H-NMR (500 MHz), ^13^C-NMR (125 MHz), Heteronuclear Multiple Bond Correlation (HMBC) experiment, COSY and NOESY data of compound **1** (CD_3_OD, δ ppm). Heteronuclear multiple-quantum correlation spectroscopy (HMQC).

-	-	^1^Hx^13^C HMQC	^1^Hx^13^C HMBC	^1^Hx^1^H COSY	^1^Hx^1^H NOESY
	δ_C_	δ_H_	-	-	-
C					-
1	154.3	-	-	-	-
5	42.3	-	-	-	-
8	156.2	-	-	-	-
9	87.0	-	-	-	-
12	47.0	-	-	-	-
14	82.1	-	-	-	-
CH					
4	81.1	3.43 (brd)	C-2, C-14	-	-
7	120.9	5.60 (dd, *J* = 4.5 and 9.5 Hz)	C-5, C-6, C-9, C-12,	-	-
17	35.2	1.99 (hept, *J* = 7.0 Hz)	C-9, C-10, C-18, C-19	-	H-7
CH_2_					
2	27.8	2.89 (dt, *J* = 5.0 and 13.5 Hz) 2.02 (m)	C-1, C-3, C-15 C-1, C-3, C-4, C-14, C-15	H-3	-
3	32.0	1.92 (m) 1.73 (m)	-	H-2	-
6	32.5	3.33 (dd, *J* = 4.5 and 15.5 Hz) 1.67 (dd, *J* = 9.5 and 15.5 Hz)	C-7, C-8, C-14, C-16 C-4, C-5, C-7, C-8, C-14	H-7	H-7
10	29.9	1.42 (m) 1.79 (m)	C-8, C-9, C-11, C-12	-	-
11	42.3	1.79 (m) 1.42 (m)	C-20 C-8, C-9, C-12	-	-
13	45.0	1.89 (d, *J* = 14.5 Hz) 1.76 (d, *J* = 14.5 Hz)	C-11, C-12, C-14, C-20 C-11, C-12, C-14, C-20	-	-
15	109.5	4.89 (s) 4.76 (s)	C-1, C-2, C-14 C-1, C-2, C-14	H-2	-
CH_3_					
16	20.0	0.81 (s)	C-4, C-5, C-6, C-14	H-4	H-4
18	17.7	1.01 (d, *J* = 7.0 Hz)	C-9, C-17, C-19	H-17	-
19	19.5	0.83 (d, *J* = 7.0 Hz)	C-9, C-17, C-18	H-17	-
20	24.5	1.22 (s)	C-8, C-11, C-12, C-13	-	H-6

**Table 2:** Chemical composition (%) of the saponifiable fatty acids from *Padina sanctae-crucis* Børgesen, obtained by trans-esterification of the 1/8 fraction from the hexane phase [27].

Saturated Fatty Acids (%)	Unsaturated Fatty Acids (%)
myristic acid (6.02)	oleic acid (1.61)
palmitic acid (68.84)	linolenic acid (9.75)
stearic acid (2.87)	linoleic acid(0.73)
Total Saturated fatty acids = 77.73%	Total Unsaturated fatty acids = 12.09%

Total identified = 89.82%.

**Table 3:** Effects of methanolic extract (ME) (2000 mg/kg) on water, feed consumption and weight gain for the experimental mice.

Groups	Sex	Dose (mg/kg)	Water Consumption (mL)	Feed Consumption (g)	Initial Weight (g)	Final Weight (g)
Control	M	-	54.82 ± 2.00	43.71 ± 1.2	32.32 ± 1.13	38.42 ± 1.54
	F		49.64 ± 1.23	41.75 ± 1.14	34.70 ± 1.20	34.42 ± 1.06
ME	M	2000	52.86 ± 2.08	39.90 ± 0.89	35.55 ± 0.56	39.16 ± 1.76
	F		47.86 ± 1.63	39.29 ± 1.16	35.40 ± 0.67	34.97 ± 1.33

Data are presented as means ± standard error of mean.

**Table 4:** Effects of methanolic extract (ME) (2000 mg/kg) on organ weights of the experimental mice.

Groups	Sex	Dose (mg/kg)	Heart (mg/g)	Liver (mg/g)	Kidneys (mg/g)	Thymus (mg/g)	Spleen (mg/g)
Control	M	-	3.92 ± 0.19	65.25 ± 2.20	13.58 ± 0.57	4.68 ± 0.30	2.17 ± 0.16
	F		4.38 ± 0.30	63.34 ± 5.54	11.72 ± 0.69	4.40 ± 0.27	2.80 ± 0.32
ME	M	2000	3.92 ± 0.12	61.84 ± 2.00	12.86 ± 0.61	4.78 ± 0.31	1.95 ± 0.40
	F		4.23 ± 0.21	57.81 ± 3.32	11.38 ± 0.49	5.11± 0.47	3.54 ± 0.34

Data are presented as mean ± standard error of the mean.

**Table 5:** Effects of methanolic extract (ME) on the biochemical peripheral blood parameters of the mice.

Groups	Sex	Aspartate Aminotransferase (U/L)	Alanine Aminotransferase (U/L)	Urea (mg/dL)	Creatinine (mg/dL)
Control	M	169.80 ± 9.31	51.00 ± 4.16	40.75 ± 2.29	0.21 ± 0.04
	F	138.80 ± 10.85	72.60 ± 19.63	49.75 ± 3.97	0.56 ± 0.07
ME	M	233.30 ± 33.36	60.33 ± 4.77	42.50 ± 1.44	0.24 ± 0.01
	F	93.67 ± 27.51	78.67 ± 23.67	50.00 ± 5.51	0.19 ± 0.02

Data presented as mean ± s.e.m. six animals.

**Table 6:** Effects of ME on the peripheral hematological blood parameters of the mice.

Parameters	Sex	Control	ME (2000 mg/kg)
Erythrocytes (10^6^/mm^3^)	M	8.59 ± 0.21	8.55 ± 0.16
	F	8.90 ± 0.26	9.58 ± 0.16 ^a^
Hemoglobin (g/dL)	M	14.32 ± 0.37	13.65 ± 0.30
	F	13.73 ± 0.64	14.62 ± 0.06
Hematocrit (%)	M	41.35 ± 1.40	40.80 ± 0.69
	F	41.67 ± 1.89	44.63 ± 1.16
MCV (fm^3^)	M	48.00 ± 0.68	47.83± 1.01
	F	45.83 ± 1.49	46.67 ± 1.15
MCH (pg)	M	16.68 ± 0.32	16.00 ± 0.44
	F	15.42 ± 0.41	15.58 ± 0.22
MCHC (g/dL)	M	34.75 ± 0.82	33.45 ± 0.45
	F	32.83 ± 0.40	33.45 ± 0.36
Total leucocytes (10^3^/mm^3^)	M	7.55 ± 0.90	14.65 ± 1.56 ^a^
	F	7.82 ± 0.47	3.98 ± 0.18 ^a^
Lymphocytes	M	70.60 ± 1.72	55.33 ± 4.70
	F	71.67 ± 4.72	75.50 ± 4.47 ^a^
Neutrophils	M	30.67 ± 4.59	41.00 ± 4.56
	F	21.83 ± 5.12	20.67 ± 3.61
Monocytes	M	3.60 ± 0.68	3.67 ± 0.72
	F	6.50 ± 0.72	4.60 ± 1.36
Eosinophils	M	1.00 ± 0.0	0.0 ± 0.0
	F	0.0 ± 0.0	0.0 ± 0.0

Data presented as mean ± s.e.m. of six animals examined by Student’s *t*-test for unpaired parametric variables, compared to the control, ^a^ *p* < 0.05. Mean corpuscular volume (MCV); mean corpuscular hemoglobin (MCH); mean corpuscular hemoglobin concentration (MCHC).

**Table 7:** Frequency of micronucleated erythrocytes in peripheral blood of mice treated with different doses of ME and cyclophosphamide.

Groups	Dose (mg/kg)	Micronucleated Cells
Control	-	2.50 ± 0.43
Cyclophosphamide	50	17.67 ± 1.38 ^a^
ME	2000	2.33 ± 0.21 ^b^

Data presented as mean ± s.e.m. of six animals analyzed by ANOVA followed by Dunnett. ^a^ *p* < 0.05 compared to control (5% Tween 80); ^b^ *p* < 0.05 compared to cyclophosphamide.
